# Education Is Treatment: Integrating Chemosensory Dysfunction Education in Oncology Care

**DOI:** 10.1007/s13187-025-02641-y

**Published:** 2025-05-02

**Authors:** Kara Stromberg, Valentina Parma, Kristen Manley, Dylan Sherry, Michael J. Hall, Alissa A. Nolden

**Affiliations:** 1 Fox Chase Cancer Center, Philadelphia, PA USA; 2 Monell Chemical Senses Center, Philadelphia, PA USA; 3 Department of Food Science, University of Massachusetts Amherst, Amherst, MA USA

**Keywords:** Taste loss, Smell loss, Cancer, Dysgeusia, Anosmia, Clinician education, Education intervention

## Abstract

Chemosensory dysfunction, defined as an altered or lost taste and smell, is a prevalent side effect of cancer treatment, with 93% of patients complaining of taste and 60% complaining of changes in smell. Despite their impact, it is an underrecognized symptom, impairing nutritional intake, quality of life, and treatment outcomes. Surprisingly, taste and smell changes are rarely assessed or addressed in oncology care. This commentary highlights the educational gap faced by clinicians and reports the results of a pilot educational intervention consisting of a 15-min podcast. The results indicate significant improvements in provider knowledge and confidence to support patients experiencing chemosensory dysfunction. By embedding chemosensory education into nutrition, survivorship, and interprofessional care pathways, clinicians can better recognize, document, and respond to these symptoms. Reframing taste and smell not as minor nuisances but as critical facets of patient well-being represents a shift toward more comprehensive oncology care.

Chemosensory dysfunction—changes or loss in taste and smell—is a highly prevalent and underrecognized side effect of cancer and its treatment. Affecting up to 93% of patients for taste and 60% for smell across cancer types, locations, and disease stages, these changes can span the disease course and persist well beyond treatment completion [1]. Far from being a minor annoyance, chemosensory dysfunction significantly impairs quality of life. It alters the flavor of all foods and beverages, diminishes social enjoyment, and raises safety concerns around hazards like gas leaks or spoiled food. Acute and persistent taste and smell dysfunction can contribute to poor dietary intake, weight loss, and malnutrition—factors associated with treatment complications and mortality [2]. Despite this, taste and smell symptoms remain largely unassessed, undocumented, and untreated, leaving patients and caregivers to navigate a complex sensory landscape alone [3].

Available patient-facing resources from patient organizations offer coping strategies (e.g., modifying cooking/eating habits), but few are evidence-based or tailored [4]. The current lack of reliable, accessible education on chemosensory effects in the oncology setting represents a missed opportunity for improving outcomes. Integrating structured education into cancer care can optimize nutritional status, reinforce therapeutic trust, and validate patients’ experiences.

## What Do Taste and Smell Changes Look Like in Oncology?

Chemotherapy, radiation, and surgical interventions can each disrupt normal taste and smell function. These disruptions are highly variable: Patients may experience reduced sensitivity, altered hedonic responses, or specific distortions such as metallic or bitter tastes [2]. During treatment, cytotoxic agents target rapidly dividing cells, including those involved in taste and smell perception. In the case of radiation to the head and neck area, patients may experience more severe changes in taste and smell. In some cases, these changes persist long after treatment has ended, reflecting lasting damage to gustatory and olfactory tissues. As Nolden et al. emphasize, the nature and timing of chemosensory dysfunction are unpredictable and highly individualized—further complicating both diagnosis and care planning [1].

## The Chemosensory Education Gap in Oncology

Taste and smell are fundamental to appetite, dietary intake, and life enjoyment more broadly. As such, their disruption should be of central concern to nurses, oncologists, dietitians, mental health professionals, speech-language pathologists, and palliative care providers. Yet, currently, there are no standardized fields in electronic health records to document such symptoms, nor are they typically queried at intake or follow-up. While clinical time is limited, we argue that screening for a symptom linked to nutritional status, mental health, and treatment adherence deserves inclusion. Early identification could enable timely nutrition interventions, psychological support, and even predictive insights into patient outcomes.

## Chemosensory Education as Oncological Treatment

Despite their relevance, taste and smell remain marginalized in oncology education and across other disciplines. This was evidenced by the delay in recognizing smell and taste dysfunction as a cardinal symptom of COVID-19 infection. Major oncology texts and training programs devote little to no attention to chemosensory health. For instance, the 690-page *Oncology Nutrition for Clinical Practice* includes just one page on this topic. National conferences such as the Academy of Nutrition and Dietetic’s Annual Food and Nutrition Conference and Expo (FNCE) and the American Society of Clinical Oncology (ASCO) annual conference have not offered any sessions on chemosensory dysfunction in recent years.

**Fig. 1:**
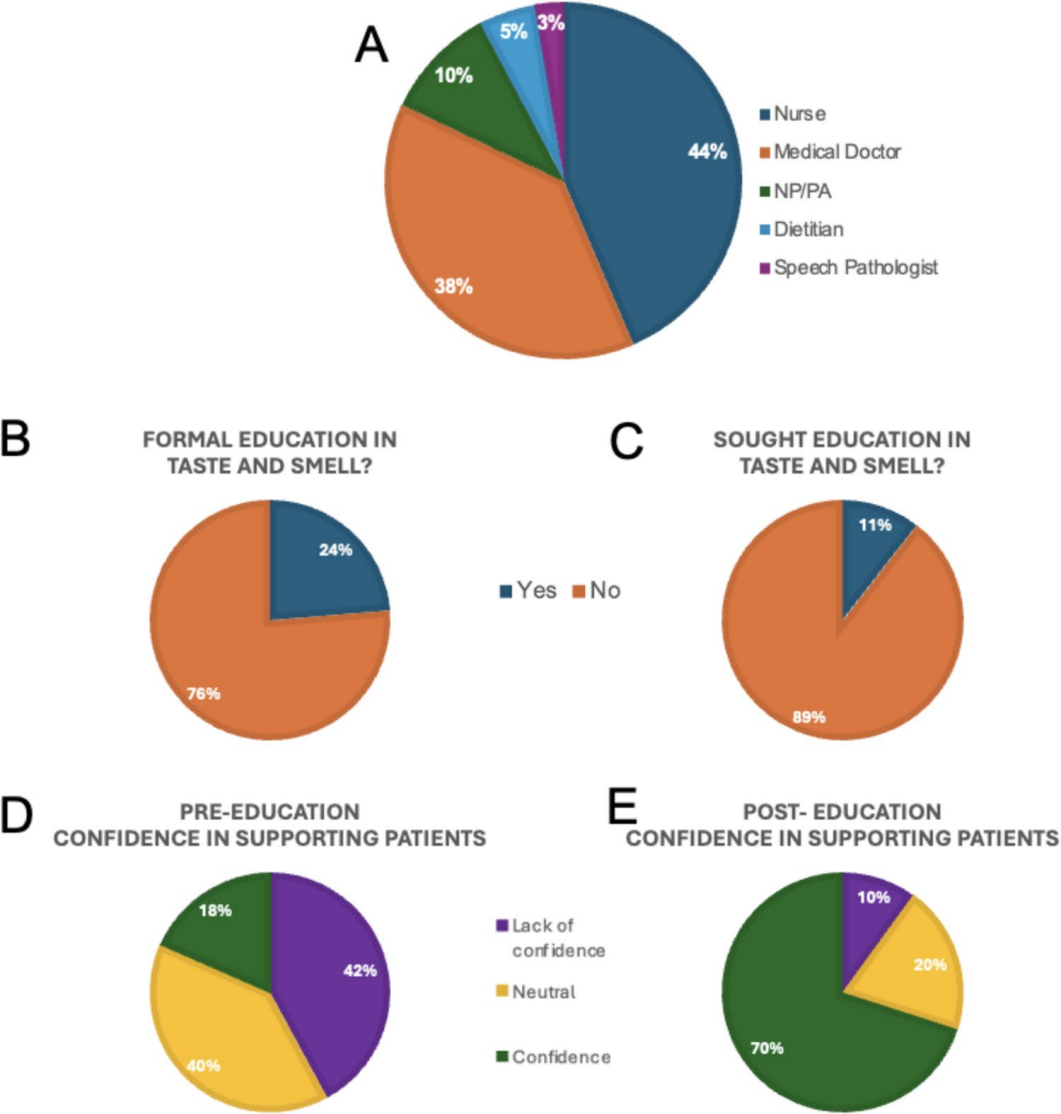
Data from a pilot study of 38 clinicians in an oncology clinic with diverse medical backgrounds (**A**) shows a lack of formal (**B**) and spontaneous (**C**) education in taste and smell and low confidence in supporting cancer patients with chemosensory issues (**D**). After completing a 15-min educational podcast on chemosensation, clinicians reported a four-fold increase in confidence in supporting cancer patients with chemosensory issues (**E**). *Note*: NP/PA: nurse practitioner/physician assistant

The result? Most clinicians receive minimal to no training. In our recent pilot study, 38 oncology clinicians (Fig. 1A) reported limited knowledge of and education in chemosensory function (Fig. 1 B and C) and low confidence in managing taste and smell changes (Fig. 1D). Many adopted a “wait-and-see” approach, citing the lack of pharmacological treatments. Yet, these same providers recognized the profound impact of these symptoms on patients’ quality of life and nutrition (Fig. 1).

Patients often turn to online forums or non-evidence-based materials for guidance [5]. Even well-meaning resources like the American Cancer Society’s “Treating Taste and Smell Changes” provide only general suggestions like “try seasoning your food more” without tailoring advice to specific symptoms and perpetuating the idea that taste and smell are negligible senses. This further supports downplaying the challenging task of eating when foods and beverages taste like cardboard or have no taste, during a time when nutritional intake and weight are critical to clinical outcomes. This generic approach fails to address individual needs, reduces patient trust, and erodes the clinician-patient relationship.

Education can change this dynamic. Structured training can improve clinician confidence, enhance patient support, and reinforce therapeutic bonds. The goal is not merely to restore taste and smell but to prevent downstream consequences such as malnutrition, reduced adherence, and emotional distress.

Education in chemosensation need not be burdensome. A brief, a 15-min podcast featuring chemosensory experts effectively introduces key concepts—including the distinction between taste, smell, and flavor; the limitations of patient self-reports in chemosensation; and the wide-ranging impact of chemosensory dysfunction on daily life and quality of life. When shared with healthcare professionals across oncology care settings, this accessible format significantly improves provider confidence in addressing taste and smell dysfunction (Fig. 1D–E), while also addressing a critical educational gap that is rarely filled spontaneously in clinical training. Structured educational efforts, particularly those that are accessible and flexible, such as those delivered via podcast, demonstrate that targeted, evidence-informed content can shift provider awareness and response to symptoms, holding promise to ultimately enhance patient care.

## Looking Ahead

Incorporating chemosensory education into oncology care is both feasible and impactful. Educational programming can be embedded into existing nutrition or survivorship pathways, offered through interprofessional teams, or delivered via digital platforms. Standardized screening questions would empower providers to address these symptoms systematically and improve clinician confidence, patient trust, and outcomes. Chemosensory perception is more than a side effect—it is a gateway to quality nutrition, emotional well-being, and dignity during treatment. As the cancer care community continues to embrace patient-centered education, taste and smell must no longer be the forgotten senses.

## Data Availability

Data will be made available upon request to the corresponding author.
